# Multifaceted quorum-sensing inhibiting activity of 3-(Benzo[d][1,3]dioxol-4-yl)oxazolidin-2-one mitigates *Pseudomonas aeruginosa* virulence

**DOI:** 10.1080/21505594.2025.2479103

**Published:** 2025-03-19

**Authors:** Yi Wu, Fulong Wen, Shiyi Gou, Qiman Ran, Yiwen Chu, Wenbo Ma, Kelei Zhao

**Affiliations:** Antibiotics Research and Re-Evaluation Key Laboratory of Sichuan Province, School of Pharmacy, Chengdu University, Chengdu, Sichuan, China

**Keywords:** Anti-virulence drugs, inhibitor, oxazolidinone, *Pseudomonas aeruginosa*, quorum-sensing, virtual screening

## Abstract

As antibiotic resistance escalates into a global health crisis, novel therapeutic approaches against infectious diseases are in urgent need. *Pseudomonas aeruginosa*, an adaptable opportunistic pathogen, poses substantial challenges in treating a range of infections. The quorum-sensing (QS) system plays a pivotal role in orchestrating the production of a large set of virulence factors in a cell density-dependent manner, and the anti-virulence strategy targeting QS may show huge potential. Here, we present a comprehensive investigation into the potential of the synthesized compound 3-(benzo[d][1,3]dioxol-4-yl)oxazolidin-2-one (OZDO, C_10_H_9_NO_4_) as a QS inhibitor to curb the virulence of *P. aeruginosa*. By employing an integrated approach encompassing *in silico* screening, *in vitro* and *in vivo* functional identification, we elucidated the multifaceted effects of OZDO. Molecular docking predicted that OZDO interfered with three core regulatory proteins of *P. aeruginosa* QS system. Notably, OZDO exhibited significant inhibition on the production of pyocyanin, rhamnolipid and extracellular proteases, biofilm formation, and cell motilities of *P. aeruginosa*. Transcriptomic analysis and quantitative real-time PCR displayed the down-regulation of QS-controlled genes in OZDO-treated PAO1, reaffirming the QS-inhibition activity of OZDO. *In vivo* assessments using a *Caenorhabditis elegans*-infection model demonstrated OZDO mitigated *P. aeruginosa* pathogenicity, particularly against the hypervirulent strain PA14. Moreover, OZDO in combination with polymyxin B and aztreonam presented a promising avenue for innovative anti-infective therapy. Our study sheds light on the multifaceted potential of OZDO as an anti-virulence agent and its significance in combating *P. aeruginosa*-associated infections.

## Introduction

While bacteria continue to develop antibiotic resistance, the pipeline of new antibiotics has encountered a drought. Valuable lessons can be gleaned from the discovery of penicillin by Alexander Fleming [1]. Furthermore, organisms exhibiting specific resistance to upcoming antibiotics, such as vancomycin, methicillin, cefotaxime, and others, were identified in subsequent years [2]. The most recent report from the World Health Organization (WHO) designates antimicrobial resistance – encompassing bacteria, viruses, fungi, and parasites – as one of the top 10 global public health threats. This is primarily due to the misuse and overuse of antimicrobial agents [3]. Adding to the severity, the review on antimicrobial resistance published by the UK government argued that antimicrobial resistance (AMR) could potentially claim the lives of up to 10 million people annually by 2050 [4]. Governments worldwide have been diligently working to curtail and prevent the development of resistance. Among these efforts, the pursuit of discovering new drugs holds exceptional promise.

Instead of directly killing bacteria like conventional antibiotics, an alternative strategy involves targeting virulence factors that are necessary for inducing host damage and promoting disease during infection was increasingly being explored [5]. Virulence factors encompass virulence determinants (such as toxins, cytolysins, proteases, etc.) or mechanisms that inflict harm on host tissues and evade the host immune system. In contrast to antibiotics, anti-virulence drugs exert reduced evolutionary pressure for resistance development and have milder impact on the host’s commensal flora [5,6]. One of the most extensively investigated avenues for anti-virulence therapy is to inhibit the quorum-sensing (QS) system, a form of bacterial cell-to-cell communication identified in the marine bacterium *Vibrio fischeri* [7,8]. Typically, QS systems operate through the synthesis and sensing of small, diffusible signal molecules, also referred to as autoinducers. In one of the most straightforward instances of QS discovered in *Vibrio fischeri*, LuxI synthesizes acylated homoserine lactones (AHLs), while LuxR detects AHLs when these autoinducers attain a critical concentration due to the elevated density of bacterial cells. This dual interplay of releasing and sensing QS signals enables the regulation of gene expression tied to virulence factors, enabling individual cells to both assess and gauge the magnitude of their community [9].

Recent research has demonstrated that anti-virulence therapy targeting QS has the potential to mitigate the pathogenicity of antimicrobial-resistant bacteria [10]. *Pseudomonas aeruginosa*, an opportunistic pathogen and a key model organism in studying bacterial QS, stands as a primary cause of hospital-acquired infections in both abiotic and biotic environments [11]. The pathogenic attributes of *P. aeruginosa* are closely linked to cell adhesion and motility facilitated by flagella and pili, biofilm formation, enzyme secretion, exotoxin production, siderophore synthesis, rhamnolipid release, hydrogen cyanide production, and the secretion of lectins. Biofilm formation is considered a key role in the persistence of *P. aeruginosa* in chronically infected lungs. Biofilm-related bacterial cells are significantly more resistant to antibiotics and host immune defence and thus are harder to be eradicated than their counterparts [12,13]. Multiple elements are proved to be involved in biofilm formation processes, such as adhesins, type IV pili and lipopolysaccharide that are crucial to the biofilm attachment [14]. Alginates and rhamnolipids are known as important virulence factors, while the former are the main constituent of biofilm and the latter mediate the active dispersal of cells from the biofilm and the passage of liquid and nutrients within mature biofilm [15,16]. Other factors such as motility, enzymes, exotoxins, and protein secretion systems are related not only to the biofilm but also impact the communities and pathogenicity of *P. aeruginosa* [17]. These virulence factors are directly or indirectly orchestrated by *P. aeruginosa* through the modulation of QS mechanisms [18].

**Figure 1.**
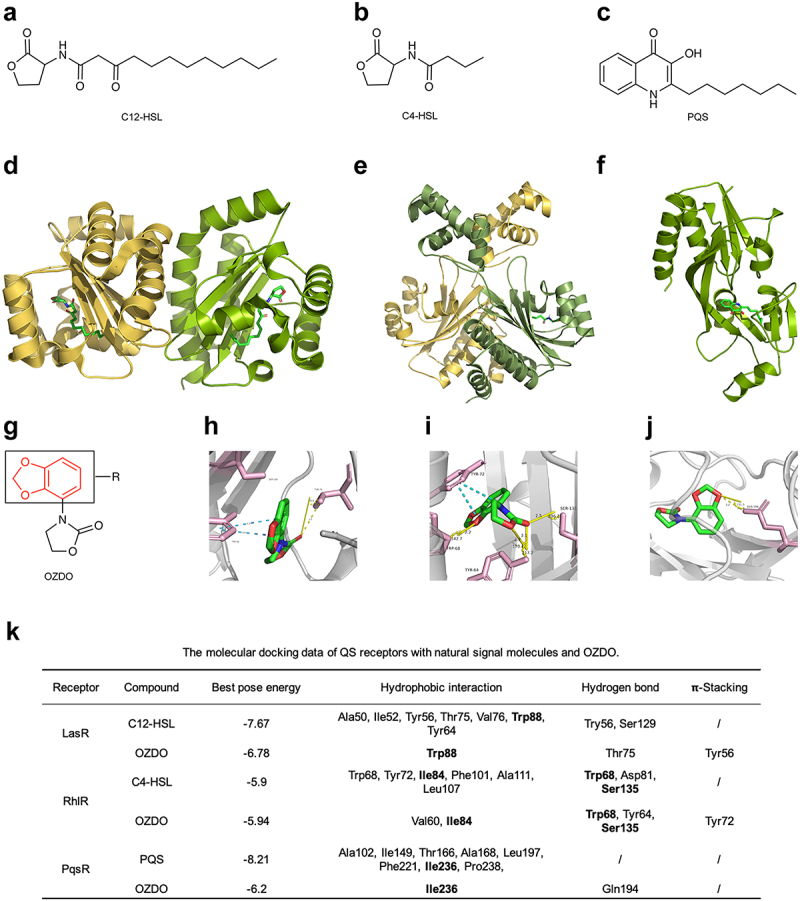
Structures of compound and *Pseudomonas aeruginosa* QS signal molecules and the molecular docking data. a QS signal molecule C12-HSL. b QS signal molecule C4-HSL. c QS signal molecule PQS. d Topology of LasR and the ligand binding domain (LBD). e Topology of RhlR and the LBD. f Topology of PqsR and the LBD. g The structure of OZDO. h 3D visualization of OZDO-LasR complex, π-stacking interaction (blue), and hydrogen bonds with parameters. i 3D visualization of the OZDO-RhlR complex, π-stacking interaction (blue), and hydrogen bonds with parameters. j 3D visualization of the OZDO-PqsR complex and hydrogen bonds with parameters. k The molecular docking data of QS receptors with natural signal molecules and OZDO.

The *las* and *rhl* QS systems, facilitated by the respective autoinducers OdDHL (*N*-(3-oxododecanoyl)-homoserine lactone, C12-HSL) (Figure 1a) and BHL (N-butyryl homoserine lactone, C4-HSL) (Figure 1b), oversee a broad spectrum of QS-responsive gene expression, with *las* occupying a prominent position. Together, these systems constitute approximately 10% of the *P. aeruginosa* genome [19]. The LuxI homologs, LasI and RhlI, are responsible for autoinducer production, while the LuxR homologs, LasR and RhlR, serve as receptors. The function of signal molecules is akin to transcription factors, and the resultant autoinducer-receptor complexes bind to conserved regions within the promoters of target genes. Another significant QS system, *pqs*, governed by its signal molecule PQS (2-heptyl-3-hydroxy-4-quinolone) (Figure 1c), is also co-regulated by the *las* system [20,21]. Consequently, the intricate QS networks orchestrate the transcriptional activation of virulence genes [19]. Disrupting QS can be achieved through various mechanisms, such as the design of antagonists of AI receptors or inhibitors of the enzymes involved in their biosynthesis, the capture of AIs by antibodies or other macromolecules such as cyclodextrins and the enzymatic degradation of extracellular AIs [18]. The AIs degradation of Quorum Quench (QQ) methods was tested to be more effective for clinical isolates which harbour QS-mutations frequently [22,23]. However, quorum sensing inhibitors (QSIs) are more studied since numerous natural or synthetic QSIs have been found from the abundant chemical sources. Notably, furanones, azithromycin, and garlic extract containing the active QSI ajoene have been the focus of substantial research to date [24].

In this study, we identified a novel compound through virtual screening based on the Oxazolidinone core structure and the three core regulators of the *P. aeruginosa* QS system. Among 37 compounds which vary in R-group, we found one may exhibit potential for anti-virulence effects. The structure of the compound, 3-(benzo[d][1,3]dioxol-4-yl)oxazolidin-2-one (OZDO, C_10_H_9_NO_4_), features a benzo-oxa-heterocycle linked to the third position of the oxazolidinone core (Figure 1d–j). The anti-virulence activity of OZDO was then verified by a series of phenotypic identification experiments, followed by RNA-sequencing. Furthermore, OZDO could protect *Caenorhabditis elegans* from *P. aeruginosa* challenge, especially in the slow-killing assay mimicking a chronic infection condition, and remarkably promote the bactericidal activity of polymyxin B and aztreonam.

## Materials and methods

### Bacterial strains and media

The model *P. aeruginosa* strains PAO1 and PA14 and clinical strains utilized in this study were preserved within our laboratory [25,26]. The media were Lysogeny broth (LB), Müller–Hinton broth (MH), M9 minimum growth medium [27] supplemented with 0.1% (*wt/v*) adenosine or 0.5% (*wt/v*) skim milk powder, Slow Killing agar medium (SK) and Peptone-Glucose-Sorbitol agar medium (PGS) [28]. Overnight cultures from a single colony were grown in LB, and their optical density at 600 nm (OD_600_) was adjusted to 1 using saline for subsequent procedures.

### Molecular docking

The crystal structures of LasR (3IX3) (Figure 1d) and PqsR (6B8A) (Figure 1f) were obtained from the Protein Data Bank (PDB). The structure of RhlR (P54292) (Figure 1e) was retrieved from the AlphaFold Protein Structure Database. Subsequent optimization was performed using PyMol (version 2.5.5). The docking of 37 compounds with the oxazolidinone core structure and the native QS signal molecules C12-HSL, C4-HSL, and PQS with the three proteins was carried out using Dockey [29], a tool built upon Autodock4 and Autodock Vina. Each compound was docked for 10 iterations to obtain an energy-minimized best pose. The 3D visualization mapped by Pymol of the molecular docking outcomes facilitated the analysis of interactions between ligands and receptors, along with assessment of docking energy. The interaction patterns of a ligand and receptor are determined with a protein-ligand Interaction Profiler (PLIP) and displayed in PyMOL [30]. The Root Mean Square Deviation (RMSD) was calculated by Pymol.

### Quorum-sensing selective medium assay

To assess OZDO’s QS inhibitory effect, we used M9 minimal growth medium supplemented with 0.1% (*w/v*) of adenosine, which is taken up and degraded by QS-controlled enzyme Nuh [31]. PAO1 (OD_600_ = 1) was cultivated (1:100) in 200 μL of LB broth or M9-adenosine medium supplemented with different concentrations of OZDO (0 µM, 25 μM, 50 μM, 100 μM, and 200 μM) at 37°C for 24 hours without shaking. The cell densities were monitored by measuring the absorbance at OD_600_. All the experiments were independently repeated for 3 times.

### Phenotypic identification

Pyocyanin production assay utilized absorbance measurement under pH-controlled conditions, considering the pH-sensitive nature of pyocyanin’s characteristic absorption spectrum [32]. PAO1/PA14 (OD_600_ = 1) was cultivated (1:100) in 2 mL of LB medium supplemented with different concentrations of OZDO (0 µM, 25 μM, 50 μM, 100 μM, and 200 μM) at 37°C with shaking (220 rpm). After 24 hours, the cultures were centrifuged for 2 min (12000 rpm). The supernatants were collected and followed by a two-step sequential extraction involving 1.2 mL of chloroform and 300 µL of 0.2 N HCl. The resulting HCl layer was gathered in a fresh 1.5 mL centrifuge tube and centrifuged (12000 rpm) for 5 minutes. Next, 200 µL of the upper HCl layer was transferred to a new 96-well plate, and the OD_520_ was measured. The amount of pyocyanin produced was calculated by multiplying the A_520_ values by a factor of 17.072, as reported previously [33]. Bacterial cells were resuspended by 1 mL of saline and the cell density was measured at OD_600_.

Elastase assay was performed by absorbance measurement as previously described [34]. PAO1/PA14 was cultivated in the same way as the pyocyanin assay above and supplemented with different concentrations of OZDO. After 24 hours, the supernatant was collected through 2-min centrifugation (12000 rpm) and was added into a new 15 mL tube with 10 mg/mL of Elastin-Congo Red in 2 mL of reaction buffer (1 mm CaCl_2_, 0.1 M Tris-HCl buffer, pH 7.0). After 3 h incubation at 37°C, the mixture was centrifuged at 12,000 rpm for 1 min and OD_495_ was measured, which was used to estimate elastase in relative quantification.

Rhamnolipid assay was measured by absorbance measurement as previously described [34]. PAO1/PA14 was cultivated in the same way above with different concentrations of OZDO. After 24 h, the supernatant was collected through 5-min centrifugation (12000 rpm) and was mixed with an equal volume of ethyl acetate. After vigorous vortex and centrifugation, the organic phase was collected and dissolved in 4 mL of chloroform added with 200 µL of freshly prepared methylene blue solution (1 g/L, pH 8.6), followed by measuring the absorbance at OD_638_ which was used to estimate rhamnolipid in relative quantification. All the experiments were independently repeated for 3 times.

Protease activity assessment employed M9-skim milk (0.5%, *w/v*) agar plates as previously described [35], and plates were supplemented with 0 µM, 25 μM, 50 μM, 100 μM, and 200 μM of OZDO. PAO1/PA14 (OD_600_ = 1) was inoculated (2 µL) onto the surface of agar plates, with 3 distinct spots on each plate. Incubation of plates was carried out at 37°C. Proteolytic zone diameters were measured after 24 hours. All the experiments above were independently repeated for 3 times.

### Biofilm formation assay

Biofilm quantification employed a crystal violet assay within 96-well polystyrene plates [36]. PAO1/PA14 (OD_600_ = 1) was cultivated (1:100) in 200 μL of LB medium supplemented with OZDO (0 µM, 25 μM, 50 μM, 100 μM, and 200 μM) for 1 to 7 days at 37°C without shaking. After measuring the cell densities at OD_600_, the culture liquids were removed, and the wells were washed by phosphate buffer solution (PBS) for 3 times. The attached biofilms were stained with 0.01% (*wt/v*) of crystal violet (220 μL) for 20 minutes. Following crystal violet removal, wells underwent 3 PBS washes. Crystal violet within attached biofilms was dissolved using 95% (*v/v*) ethanol (200 μL). Subsequently, 100 μL of the solution was transferred to a new 96-well plate, and the OD_595_ was measured [37]. All the experiments were independently repeated 3 times.

### Motility assay

Swimming, swarming, and twitching motilities of *P. aeruginosa* were assessed by using LB medium containing 0.3%, 0.5%, and 1% of agar and supplemented with 200 µM OZDO as described elsewhere [38,39]. PAO1/PA14 (OD_600_ = 1) was inoculated (2 µL) at the central area of plates using a sterile pipette tip and immersed into the middle of swimming plates and the bottom of twitching plates. Each experiment included a DMSO-negative control and performed in triplicate. Plates were incubated at 37°C for 24 hours. Crystal violet was employed to visualize the diffuse interstitial zones characteristic of twitching motility.

### Transcriptomic analysis

Total RNA samples from both OZDO-treated and untreated PAO1 were collected using the same q-PCR assay method. RNA-sequencing (RNA-seq) was conducted using the Illumina NovaSeq 6000 platform (Novogene Bioinformatics Technology, China). The obtained reads with high quality were aligned to the reference genome of *P. aeruginosa* PAO1 (NCBI accession No.: AE004091.2) using Bowtie2 (version 2.3.4.3). DESeq 2 and HTSeq (v0.9.1) were used to quantify the gene expression level and calculate the Fragments Per Kilobase of transcript sequence per millions base pairs sequenced (FPKM). *p* values were adjusted using the Benjamini & Hochberg method and padj < 0.05 and |log2(foldchange)| > 0 were set as the threshold for significantly differential expression. The ClusterProfiler R package (version 3.8.1) was used to conduct Gene Ontology (GO) enrichment analysis of differentially expressed genes and to assess the statistical enrichment of these genes in KEGG pathways.

### Quantitative real-time PCR

TRIzol reagents (Thermo) and the Total RNA Isolation Kit with gDNA removal (Foregene Biothchnology Co. Ltd, China) were employed to extract total RNAs from both OZDO (200 µM)-treated and untreated PAO1 samples. Quantitative real-time PCR was conducted using iTaqTM universal SYBR Green Supermix (Bio-Rad) and the CFX Connect Real-Time PCR Detection System. The expression levels of typical genes positively controlled by the QS system of PAO1, including *lasB*, *lasR*, *rhlA*, *rhlR*, *pqsA*, *pqsR*, *phzA*, and *hcnA* (Table S1), were validated by using 16s rRNA as the internal reference. Gene expression levels were determined by utilizing the 2^−∆∆ct^ method.

### *Caenorhabditis elegans* killing assay

The fast-killing assay was conducted on PGS agar medium, while the slow-killing assay was carried out using SK agar medium as previously described [33,40]. Thin lawn plates were created by spreading 20 µL of PA14 or PAO1 (OD_600_ = 1) with 200 µM OZDO. The plates were incubated first at 37°C for 24 hours and then at 25°C for another 24 hours. Each experiment consisted of a DMSO negative control and an uninfected control using *Escherichia coli* OP50. All experiments were conducted in triplicate. In the killing assays, 10 age-matched L4 stage nematodes were added to each prepared assay plate. The plates were subsequently incubated at 25°C.

### Antibiotic synergy test

Synergy effects of OZDO combined with aztreonam, polymyxin B, and levofloxacin on *P. aeruginosa* PAO1 and clinical isolates were evaluated through the checkerboard assay [41,42]. PAO1 was inoculated in MH broth to reach a final CFU of 10^5^ mL^−1^. OZDO (0 µM, 12.5 µM, 25 µM, 50 µM, 100 µM, and 200 µM) was then combined with antibiotics across a range of concentrations. Each combination was performed in triplicate. Incubation of the 96-well plate was carried out at 37°C for 16 hours, followed by the measurement of OD_600_. The identical procedure was applied for synergy tests involving aztreonam-resistant clinical isolate 7-R4-24, polymyxin B-resistant clinical isolate 7-61-28, and levofloxacin-resistant clinical isolate 3-100-1. The degree of synergy (S) was calculated using the Bliss independence model [43], employing the formula S = f_X,0_ · f_0,Y_ = f_X,Y_. A value of S = 0 signifies independent drug actions. Conversely, S < 0 implies an antagonistic interaction, while S > 0 suggests synergy.

### Statistical analysis

The creation of graphs and the analysis of significance, including one-way analysis of variance (ANOVA), two-sample t-test, and Log-rank test, were performed using GraphPad Prism (version 9.5.1). Significance was denoted by a *P*-value below 0.05, signifying differences between the treated and control groups.

## Results

### Molecular docking of OZDO and QS receptor proteins

Molecular docking was employed to analyze the potential interactions of the 37 compounds with QS receptors LasR, RhlR, and PqsR of *P. aeruginosa*. LasR and RhlR are LuxR-type transcription factors of *las* and *rhl* circuits, respectively, and all functional LuxR homologues contain an acyl-HSL-binding region and a helix-turn -helix motif in their carboxyl terminus [44]. A monomer fold of LasR (Figure 1d) is an α-β-α sandwich with 3 α-helices packed on both sides of a five-stranded anti-parallel *β*-sheet, and the ligand-binding-domain locates at Met-1 to Lys-173 [45]. The putative RhlR structure (Figure 1e) consists of an N-terminal ligand-binding-domain and a C-terminal helix-turn-helix DNA-binding-domain [46]. PqsR, a LysR-type transcription factor of the *pqs* circuit, has typical two *α*/*β* sub domains connected by an anti-parallel *β*-sheet known as the hinge region (Figure 1f) [47,48].

Like the binding status of the native autoinducers C12-HSL, C4-HSL, and PQS, OZDO was predicted to be capable of simultaneously binding to the ligand binding domain (LBD) of LasR, RhlR, and PqsR (Figure 1d–j). Notably, OZDO engaged in hydrogen bond interactions with amino acid residues Thr75 of LasR (Figure 1h); Tyr64, Trp68, and Ser135 of RhlR (Figure 1i); and Gln194 of PqsR (Figure 1k). Compared to the interaction pattern of native ligands, less hydrophobic interactions and hydrogen bonds were found in the OZDO-LasR complex than in the C12-LasR complex (Figure S1). However, OZDO engaged in π-stacking with Tyr56 (Figure 1h) on account of the conjugated system within the OZDO structure. OZDO was similar with C4-HSL in forming hydrogen bonds with RhlR receptor (Figure S1), and the docking energy of OZDO with RhlR was lower than of C4-HSL (Figure 1k). The hydrophobic interactions exhibited by OZDO with RhlR were less pronounced compared to C4-HSL; hence, the π-stacking interaction might contribute to reducing the docking energy. Similarly, the hydrophobic interactions exhibited by OZDO with PqsR were less pronounced compared to PQS, while one hydrogen bond was found in the docking complex of OZDO (Figure 1j).

### OZDO inhibits the qs-related phenotypes of *P. aeruginosa*

**Figure 2.**
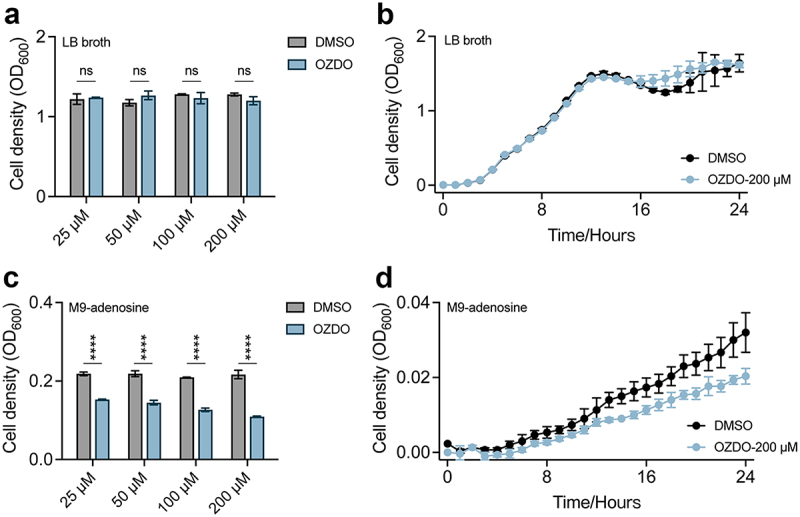
Growth status of *P. aeruginosa* PAO1 under different culture conditions. a Cell densities of *P. aeruginosa* PAO1 cultured in LB broth supplemented with different concentrations OZDO for 24 h. b Growth curve of PAO1 in LB broth supplemented with 200 µm of OZDO. c Cell densities of *P. aeruginosa* PAO1 cultured in M9-adenosine supplemented with different concentrations OZDO for 24 h. d Growth curve of PAO1 in M9-adenosine supplemented with 200 µm of OZDO. Data shown were means ± standard deviation (SD) of three independent replicates. One-way ANOVA test compared to the control group. ****, *p* < 0.0001.

The minimal inhibitory concentration (MIC) of OZDO against PAO1/PA14 was assessed first, revealing a value exceeding 4000 µM (Figure S2). Because the concentrations of QS molecules in *P. aeruginosa* may vary profoundly according to growth status and conditions, in this study, the effects of OZDO on the virulence-related phenotypes of *P. aeruginosa* was determined by using different concentrations of OZDO (0 µM to 200 µM) based on previous studies [18,49,50]. The growth of PAO1 or PA14 was significantly inhibited by the supplementation of OZDO in M9-adenosine medium (Figures. 2a,b and S3a). By contrast, the results showed that the supplementation of OZDO at the contractions ranged from 25 µM to 200 µM had no significant effect on the growth of PAO1 or PA14 in LB broth (Figures. 2c,d and S3b). M9-adenosine medium was used to preliminarily access the inhibition of OZDO on the *P. aeruginosa* QS system because the growth of *P. aeruginosa* in this medium requires the production of QS-controlled hydrolase Nuh [27]. These results suggested that OZDO had no bactericidal activity on *P. aeruginosa* but might inhibit the QS system.

**Figure 3.**
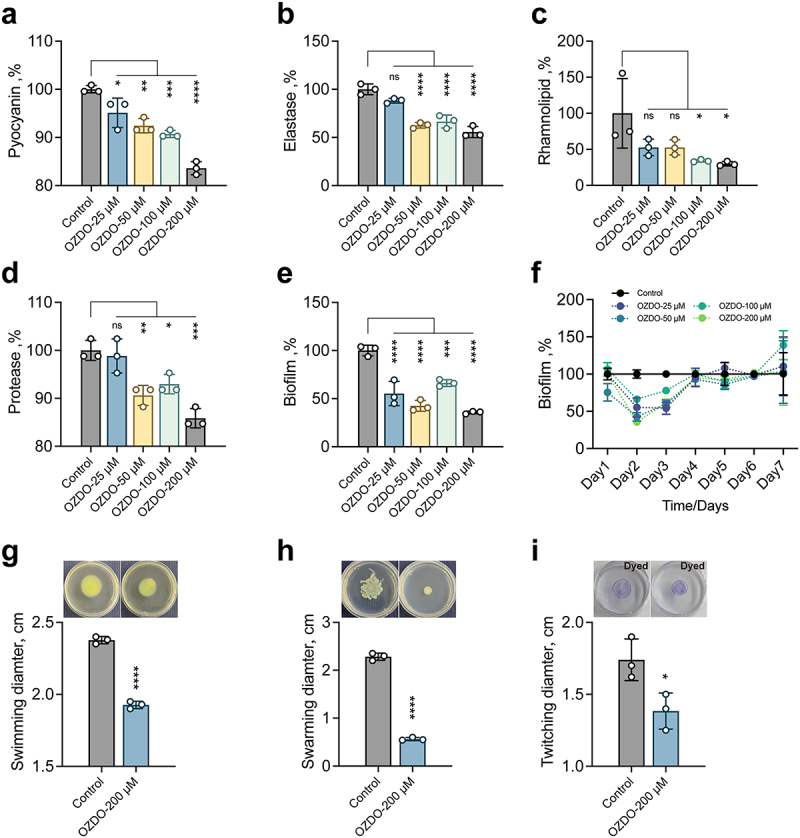
OZDO inhibits the virulence-related phenotypes of *P. aeruginosa* PAO1. a Pyocyanin assay. b Elastase assay. c Rhamnolipid assay. d Protease assay. e Biofilm formation assay in day 2. f 7-day biofilm formation assay. g Swimming motility assay of PAO1 treated with OZDO (200 µm) and quantified using the diameter (cm) of diffuse interstitial zones. h Swarming motility assay of PAO1 treated with OZDO (200 µm) and quantified using the diameter (cm) of branch width. i Twitching motility assay of PAO1 treated with OZDO (200 µm) and quantified using the crystal violet dyed diameter (cm). Data shown were means ± SD of three independent replicates. One-way ANOVA test compared to the control group. *,*p* < 0.05; **,*p* < 0.01; ***,*p* < 0.001; ****,*p* < 0.0001.

Considering that virulence factors such as pyocyanin, elastase, rhamnolipids, and proteases significantly contribute to *P. aeruginosa* pathogenicity, orchestrated by QS systems during infection, we then evaluated the influence of OZDO on the production of these QS-regulated virulence factors [51]. The results showed that OZDO showcased a dose-dependent (25 µM to 200 µM) inhibitory activity on the production of pyocyanin of PAO1, compared to the DMSO control (Figure 3a) while 200 µM of OZDO did not show inhibitory effect in pyocyanin assay of PA14 (Figure S3c). The production of elastase and rhamnolipids of PAO1 could also be inhibited by higher concentrations of OZDO (Figure 3b,c). Similar results were found in the rhamnolipid assay of PA14 while elastase assay was less prominent (Figure S3e, f). Moreover, notable inhibition of extracellular protease production of both PAO1 and PA14 was detected at higher concentrations of OZDO (Figure 3d and S3f). Therefore, these results demonstrated that the QS-related phenotypes of *P. aeruginosa* could be inhibited by the treatment of OZDO, especially when the concentration of OZDO was used at 200 µM.

### OZDO inhibits the biofilm formation and cell motilities of *P. aeruginosa* PAO1

The formation of biofilm by *P. aeruginosa* is one of the hallmarks of their survival both *in vivo* and in harsh environmental conditions; thus, biofilm inhibition is of major concern for therapeutic strategies and for infection control [52]. At day 2, the tested concentrations of OZDO (25 µM to 200 µM) displayed significant inhibition on the biofilm formation of PAO1, compared to untreated control (Figure 3e). OZDO-treatment inhibited the production of biofilm in the first three days, while the relative percentage of biofilm became comparable to that of untreated group after 4 days (Figure 3f). Due to the slower growth of PA14 than PAO1, the most significant inhibitory effect on biofilm formation displayed at day 3 and faded at day 5 (Figure S3g). We further tested the influence of OZDO on the motility of PAO1 and PA14, which is contingent upon the flagella and type IV pili and is pivotal for processes like surface contact, transitioning to a sessile lifestyle, and fostering biofilm formation [15]. Compared to the DMSO control group, OZDO at the concentration of 200 µM exhibited a noteworthy 19% decrease in the average diameters of diffuse interstitial zones pertaining to swimming motility (Figure 3g), a noteworthy 75% decrease in swarming motility (Figure 3h), and a corresponding 21% reduction in the context of twitching motility of PAO1 (Figure 3i). Similar results were found in swarming and twitching motility assay of PA14 while OZDO displayed no inhibitory effect on swimming motility of PA14 (Figure S3h-j). These results clearly demonstrated the inhibitory activity of OZDO on the biofilm formation and cell motilities of *P. aeruginosa* PAO1 and PA14. Moreover, based on the results of phenotypic identification above (Figure 2 and 3), 200 µM was regarded as the optimal working concentration of OZDO in the subsequent experiments.

### OZDO down-regulates genes activated by *P. aeruginosa* QS system

**Figure 4.**
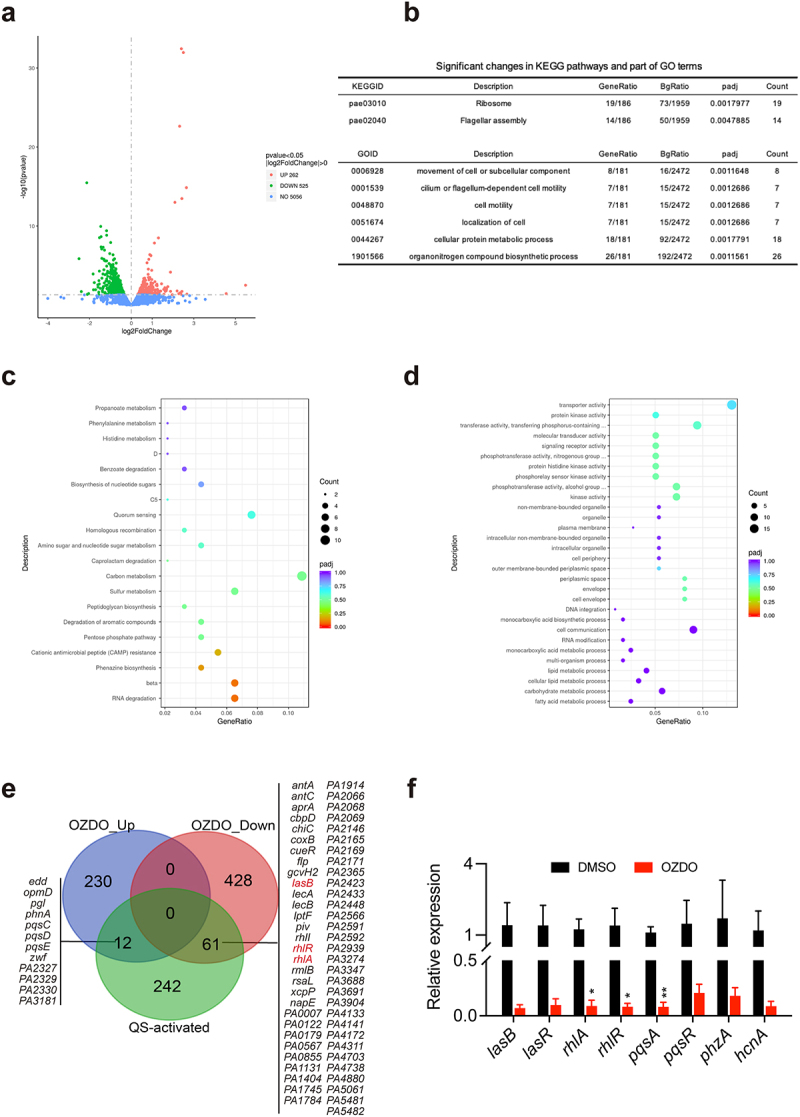
Effect of OZDO on the transcriptome of *P. aeruginosa* PAO1. a The volcano plot of significantly differentially expressed genes of PAO1 influenced by 200 µm of OZDO compared to DMSO group. b Significantly down-regulated KEGG and partial GO terms. The bubble diagram of significantly up-regulated KEGG (c) and GO terms (d) of PAO1 influenced by 200 µm of OZDO. e the numbers and lists of significantly differentially expressed qs-induced genes in PAO1 treated with 200 µm of OZDO. f Expression of typical qs-induced genes determined by quantitative PCR in PAO1 treated with 200 µm of OZDO. Data shown were means ± SD of three independent replicates. Unpaired two-tailed t test compared to the control group. *,*p* < 0.05; **,*p* < 0.01.

RNA-seq was then performed to assess the influence of OZDO (200 µM) on the global transcription of *P. aeruginosa* PAO1. In comparison to the untreated group, a total of 525 down-regulated genes and 262 up-regulated genes were identified in the treated group (Figure 4a). KEGG functional annotation demonstrated significant enrichment of the ribosome and flagellar assembly pathways among the down-regulated genes in the treated group (Figure 4b), while no significant enrichment of pathways among the up-regulated genes (Figure 4c). Additionally, the treatment of OZDO caused no significant enrichment of GO terms among the up-regulated genes (Figure 4d), while significantly decreased the cell motility and localization of cell related GO terms etc. among the down-regulated genes (Figure 4b). These findings suggested that OZDO significantly influenced the biological metabolic processes of *P. aeruginosa* PAO1 at the transcriptional level. Notably, OZDO down-regulated 61 out of the 315 genes positively controlled by QS (Figure 4e). The down-regulation of genes induced by QS, including typical virulence genes such as *lasB*, *rhlA*, and *rhlR*, was further confirmed through qPCR analysis (Figure 4f). As shown in the results, the relative expression of *lasB*, *lasR*, *rhlA*, *rhlR*, *pqsA*, *pqsR*, *phzA*, and *hcnA* genes in *P. aeruginosa* PAO1 were all decreased upon the treatment of OZDO, and the expression of *rhlA*, *rhlR*, and *pqsA* were significantly down-regulated.

### OZDO protects C. elegans from *P. aeruginosa* infection

**Figure 5.**
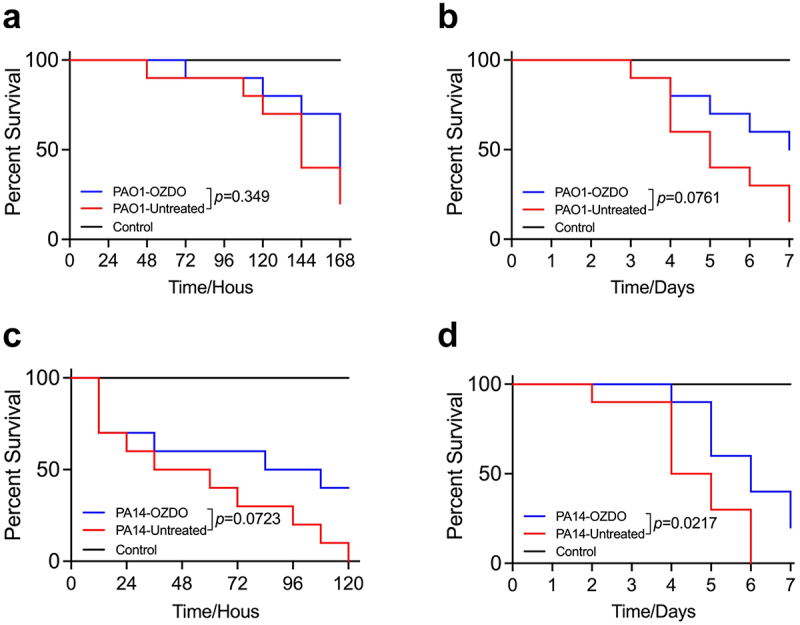
Protective effect of OZDO on *C. elegans* from *P. aeruginosa* infections. Protective effect of 200 µm OZDO on *C. elegans* infection models against PAO1 in fast (a) and slow (b) killing assay. Protective effect of 200 µm OZDO on *C. elegans* infection models against PA14 in fast (c) and slow (d) killing assays. The survival curves were compared by using Log-rank (Mantel-Cox) test.

The *C. elegans*–*P. aeruginosa* infection model was employed to investigate *the in vivo* activity of OZDO. In comparison to the uninfected group, which was inoculated with *E. coli* OP50, OZDO exhibited no toxicity to nematodes. The results showed that OZDO could increase the survival rate of *C. elegans* against *P. aeruginosa* PAO1 challenge in the slow-killing assay, which mimics a chronic infection condition, but not significantly (Figure 5a). The survival proportion was 70% at hour 144 of the OZDO treated group, while only 40% of the untreated group was 30% at hour 168 of the OZDO treated group, which was 10% higher than the untreated group. Subsequently, the results of fast-killing assay, which mimics an acute infection, showed that 50% of nematodes survived in the treated group at day 7, which was 40% higher than the untreated group (Figure 4b). To further evaluate the protective effect of OZDO, we employed the hypervirulent reference strain *P. aeruginosa* PA14 to construct the infection model. As a result, in both the fast- and slow-killing assays, the addition of OZDO (200 µM) resulted in increased survival rates of nematodes against *P. aeruginosa* PA14 infection (Figure 5c,d), especially in the slow-killing assay (*p* = 0.0217). 50% of the nematodes survived at hour 120 in the treated group, while all the nematodes died in the untreated group (Figure 5c). A significantly protective effect displayed in the slow-killing assay, and 40% of the nematodes in the treated group survived in day 6 (Figure 5d).

### Synergistic effect of OZDO and antibiotics against *P. aeruginosa*

**Figure 6.**
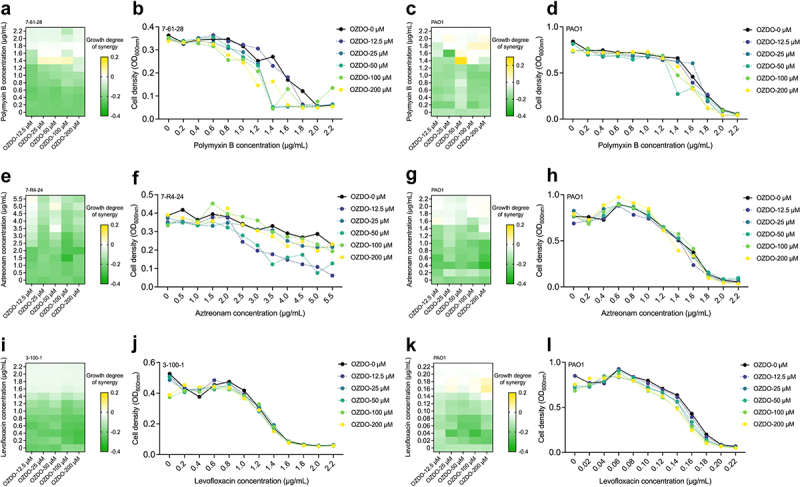
The synergic interactions of OZDO with antibiotics. a Heap map of OZDO combined with polymyxin B against clinical isolate *P. aeruginosa* 7-61-28 and the (b) growth curve. c Heap map of OZDO combined with polymyxin B against PAO1 and the (d) growth curve. e heap map of OZDO combined with aztreonam against clinical isolate *P. aeruginosa* 7-R4-24 and the (f) Growth curve. g Heap map of OZDO combined with aztreonam against PAO1 and the (h) growth curve. i Heap map of OZDO combined with levofloxacin against clinical isolate *P. aeruginosa* 3-100-1 and the (j) growth curve. k Heap map of OZDO combined with levofloxacin against PAO1 and the (l) growth curve. The yellow-green bar shows the degree of synergy calculated by the bliss independence model.

We then assessed the potential synergistic effect between OZDO and the commonly used clinical antibiotics. The MICs of aztreonam, polymyxin B, and levofloxacin against *P. aeruginosa* PAO1 and clinical isolates were determined first (Table S2), followed by examining the growth responses of *P. aeruginosa* when treated with antibiotics only or in combination with different concentrations of OZDO. The results showed that using OZDO as an adjuvant did not completely abrogate or reverse the selection for antibiotic resistance in both PAO1 and the clinical isolate. However, OZDO restored the polymyxin B susceptibility of 7-61-28 which is a polymyxin B-intermediate resistant isolate (Figure 6a,b), as the MIC of polymyxin B against 7-61-28 decreased from 2.0 µg/mL to 1.4 µg/mL when treated with 50 µM of OZDO. OZDO also restored polymyxin B susceptibility of PAO1 to some extent (Figure 6c,d) Moreover, 12.5 µM of OZDO treatment decreased the MIC of 7-R4-24 which is an aztreonam-intermediate resistant isolate to 5.5 µg/mL (Figure 6e,f) while nonsignificant synergistic effect displayed in assay of PAO1 (Figure g,f). Nevertheless, a nonsignificant synergistic effect displayed in the combination of OZDO and levofloxacin in both PAO1 and 3-100-1 which is a levofloxacin-susceptible isolate. In brief, OZDO could promote the susceptibility of polymyxin B and aztreonam against *P. aeruginosa* clinical isolates.

## Discussion

Antimicrobial resistance and persistence are associated with an elevated risk of treatment failure and relapsing infections, and ESKAPE pathogens (*Enterococcus faecium*, *Staphylococcus aureus*, *Klebsiella pneumoniae*, *Acinetobacter baumannii*, *P. aeruginosa,* and *Enterobacter* species) place a significant burden on patient health, healthcare systems, and economies [53]. *P. aeruginosa*, listed in the WHO priority group 2 of pathogens, intrinsically shows low susceptibility to many antibiotics and a high propensity to develop resistance [54,55]. Anti-virulence approaches, aiming to disarm pathogens, have emerged over the last decade and are believed to significantly reduce the selection pressure for resistant mutants [56]. For example, glycomimetics are considered potential pathoblockers, inhibiting the adhesion process [57]. Racemic phenoxyacetamide can directly inhibit the secretion of the T3SS effector ExoS in *P. aeruginosa*, and thiol-containing peptides act as potent inhibitors of metalloproteases [58]. Given that QS-controlled virulence factors normally play crucial roles in every step of the pathogenicity process, targeting QS components presents an attractive avenue for the development of anti-virulence therapies [59]. In this study, we identified OZDO through *in silico* docking as a potent QS inhibitor with the potential to serve as an anti-virulence drug.

Research has shown that the *las* system sits atop of the QS hierarchy and has been thoroughly investigated for the development of QSI [60]. Among the QS-inhibiting molecules, furanone derivatives have garnered significant attention. Both natural and synthetic furanone derivatives have demonstrated anti-QS activity as AHL autoinducer non-analog QSIs [61,62]. However, the majority of compounds targeting the *las* system alone failed during the preclinical development phase due to the complexity of QS [18]. It has been shown that there is significant phenotypic variability among clinical isolates of *P. aeruginosa*, especially strains lacking a functional *las* system, yet they are still capable of producing virulence factors [63,64]. Previous studies found that nearly 40% of *P. aeruginosa* isolates are LasR-defective strains, which occurred in clinical, environmental, or specific circumstances (a phosphate-limited growth condition); hence, the *rhl* system was suggested to play a more prominent role in ensuring QS functionality. Additionally, RhlR activity depends on PqsR-dependent regulation through the expression of *pqsE* via *pqs* operon, underscoring the importance of the *pqs* system for maintaining the full suite of QS regulation. This suggests that QS inhibitors should be multi-pronged [26,63,65–67]. Moreover, the previous study explored the combination therapy of QSI and QQ enzyme to disrupt both *las* and *rhl* AHL signal pathways, which was also a plausible way to respond to the challenge of multi-QS systems [68]. Based on the above studies, we focused on the compounds that had the possibility of targeting multiple QS systems simultaneously in virtual screening.

Interestingly, OZDO could potentially bind to QS receptors LasR, RhlR, and PqsR (Figure 1h–j). We further showed that a series of virulence factors, including biofilm formation, cell motilities, and those positively controlled by the QS system of *P. aeruginosa* were curtailed by OZDO treatment, demonstrating the practicality of this multi-pronged targeting. Compounds with similar structures, such as oxazolidinones like linezolid and tedizolid, have been used to treat infections caused by Gram-positive and some Gram-negative pathogens as antibiotics [69]. Additionally, 13 derivatives with a 3-aminooxazolidin-2-one core have been found to be potent QS inhibitors of PAO1, and the 2-oxazolidone heterocycle was considered essential for their activity [70]. Compared to the investigation of oxazolidinones conducted thus far, OZDO exhibited no growth inhibition effect on PAO1 in LB broth, unlike antibiotics (Figure 2a), and this is consistent with the characteristics of an anti-virulence drug [71]. We further evaluated the *in vivo* protective effect of OZDO against *P. aeruginosa* infection by using *C. elegans* models and the reference strain *P. aeruginosa* PAO1 and the hypervirulence strain PA14 (Figure 5). The results suggested that OZDO treatment could remarkably protect *C. elegans* from PA14 infection in the slow-killing assay. Moreover, OZDO also showed a protective effect on PA14-infected *C. elegans* in the fast killing assay and PAO1-infected *C. elegans* in both infection models, albeit the survival curves of *C. elegans* in these experiments were not significantly different from OZDO-untreated control. This might be attributed to the higher innate virulence of PA14 on *C. elegans* model than that of PAO1, and thus the anti-virulence function of OZDO against PA14 infection would be more prominent. Moreover, *P. aeruginosa*-associated acute and chronic infection models by using vertebrates may contribute to further validating the *in vivo* protective activity and revealing the detailed functional mechanism of OZDO.

Despite the advantages, anti-virulence strategies still have drawbacks. The QSI therapy may have efficiency in QS active infection but not in infection associated with non-QS controlled or negative-QS controlled pathogenicity; not to mention, the metabolism and growth process of pathogens are unaffected by QSI [72]. Hence, clearing the infection might not occur, particularly for patients with potentially compromised immune systems unable to combat even attenuated pathogens [43]. Combining anti-virulence compounds with antibiotics may serve as a strategic solution to circumvent this challenge. Ultimately, we assessed the synergistic effect of OZDO in combination with aztreonam, polymyxin B, and levofloxacin against *P. aeruginosa* PAO1 and clinical isolates, which have intact *lasR* function and are less susceptible to the corresponding antibiotic. The isolate 7-61-28 has increased capability in biofilm formation, the isolate 7-R4-24 has enhanced capability in pyocyanin production, and the isolate 3-100-1 beats the model strain PAO1 both in biofilm formation and pyocyanin production. The resistance characteristics of clinical isolates were also shown in supplementary data (Table S2). The results suggested that OZDO could increase the susceptibilities of *P. aeruginosa* clinical isolates against polymyxin B and aztreonam (Figure 6). The possible mechanism of polymyxin B synergy may be due to the down-regulation of *parR,* which results in alterations in membrane composition and down-regulation of *oprD* (porin D) and *oprG* (outer membrane protein) [73]. The down-regulation of *oprD* was also related to reducing aztreonam resistance. Moreover, *ispB* (encoding for octaprenyl-diphosphate synthase) and *PA2557* (encoding for AMP-binding protein) were both found down-regulated in the transcriptional level and also have possible roles in regulating gene expression related to aztreonam resistance [74]. These results highlighted the clinical application potential of OZDO as an adjuvant in treating infections caused by antibiotic resistant *P. aeruginosa*. However, the specific mechanism of the synergistic effect is still unclear, but it is worth further study.

In conclusion, this study reveals the multifaceted anti-virulence activity of OZDO on *P. aeruginosa*. OZDO can inhibit production of several QS-controlled extracellular virulence factors such as pyocyanin, elastase, and rhamnolipid in *P. aeruginosa*. In the meanwhile, OZDO exhibits significant inhibitory activities on the biofilm formation and cell motilities of *P. aeruginosa*. Moreover, OZDO shows a moderate protective activity on *C. elegans* models challenged by *P. aeruginosa*, especially by the hypervirulent strain PA14 in the slow-killing assay, and enhances the susceptibility of *P. aeruginosa* clinical isolates towards polymyxin B or aztreonam treatment. Therefore, these results underscore the research and development significance of OZDO and its derivatives as anti-virulence drugs or synergist of antibiotics in treating pseudomonas infections. Lastly, results suggested that OZDO deviates are worthy of further exploration, an aspect that we are currently dedicated to enhancing.

## Supplementary Material

Table S1.docx

Suppldata_v4.docx

Figure S1.tif

Figure S3.tif

Figure S4.tif

Table S2.docx

Figure S2.tif

## Data Availability

The datasets generated and analysed during the current study are available in the NCBI BioProject repository and the registration number PRJNA1017147. (https://www.ncbi.nlm.nih.gov/bioproject/PRJNA1017147/).
