# Supplementary material for: Multifaceted quorum-sensing inhibiting activity of 3-(Benzo[d][1,3]dioxol-4-yl)oxazolidin-2-one mitigates Pseudomonas aeruginosa virulence
Source: Virulence. 2025 Mar 19;16(1):2479103. doi: 10.1080/21505594.2025.2479103 (PMC12915424; doi:10.1080/21505594.2025.2479103)
Supplement: Table S1.docx [file KVIR_A_2479103_SM7656.docx]

**Multifaceted Quorum-sensing Inhibiting Activity of 3-(Benzo[d][1,3]dioxol-4-yl)oxazolidin-2-one Mitigates** ***Pseudomonas aeruginosa* Virulence**

Yi Wu, Fulong Wen, Shiyi Gou, Qiman Ran, Yiwen Chu, Wenbo Ma*, Kelei Zhao*

Antibiotics Research and Re-evaluation Key Laboratory of Sichuan Province, School of Pharmacy, Chengdu University, Chengdu 610106, Sichuan, China

* Correspondence:

Kelei Zhao, Email: zhaokelei@cdu.edu.cn

Wenbo Ma, Email: mawenbo@cdu.edu.cn

**Supplementary Tables**

**Table S1**. Primers used in this study

| Gene | Sequence (5’-3’) |
| --- | --- |
| *lasB*rtF | ATCGGCTACGACATCAAGAAGG |
| *lasB*rtR | CCGCTGTTGTAGTTGCTGGTG |
| *lasR*rtF | CTTCATCGTCGGCAACTAC |
| *lasR*rtR | GTCTGGTAGATGGACGGTTC |
| *rhlA*rtF | ACTGAACCAGGCGATGCTC |
| *rhlA*rtR | GCTCCAGGCAAGCCAAGTA |
| *rhlR*rtF | GCTCCTCGGAAATGGTGGT |
| *rhlR*rtR | GGAAAGCACGCTGAGCAAAT |
| *pqsArtF* | GCTGAGCGGTCCTTTGGC |
| *pqsArtR* | TGGAACCCGAGGTGTATTGC |
| *pqsR*rtF | CACTGGTTGAAGCGGGAGA |
| *pqsR*rtR | TCGTTCTGCGATACGGTGAG |
| *phzArt*F | GCAACTGGACCACGGAAAG |
| *phzArt*R | GCACGCAGTTTCTGTATCGG |
| *hcnArt*F | GCAGACATGACCATCCACCTC |
| *hcnArt*R | CGGTTGCTTTCGGTTTCCA |
| *16Srt*F | TCGCATCCTGTTGTCCTCCA |
| *16Srt*R | TTAGCCAGGGTCAGCGTCA |
