# Supplementary material for: Multifaceted quorum-sensing inhibiting activity of 3-(Benzo[d][1,3]dioxol-4-yl)oxazolidin-2-one mitigates Pseudomonas aeruginosa virulence
Source: Virulence. 2025 Mar 19;16(1):2479103. doi: 10.1080/21505594.2025.2479103 (PMC12915424; doi:10.1080/21505594.2025.2479103)
Supplement: Suppldata_v4.docx [file KVIR_A_2479103_SM7655.docx]

**Multifaceted Quorum-sensing Inhibiting Activity of 3-(Benzo[d][1,3]dioxol-4-yl)oxazolidin-2-one Mitigates** ***Pseudomonas aeruginosa* Virulence**

Yi Wu, Fulong Wen, Shiyi Gou, Qiman Ran, Yiwen Chu, Wenbo Ma*, Kelei Zhao*

Antibiotics Research and Re-evaluation Key Laboratory of Sichuan Province, School of Pharmacy, Chengdu University, Chengdu 610106, Sichuan, China

* Correspondence:

Kelei Zhao, Email: zhaokelei@cdu.edu.cn

Wenbo Ma, Email: mawenbo@cdu.edu.cn

**Supplementary Figures**

**
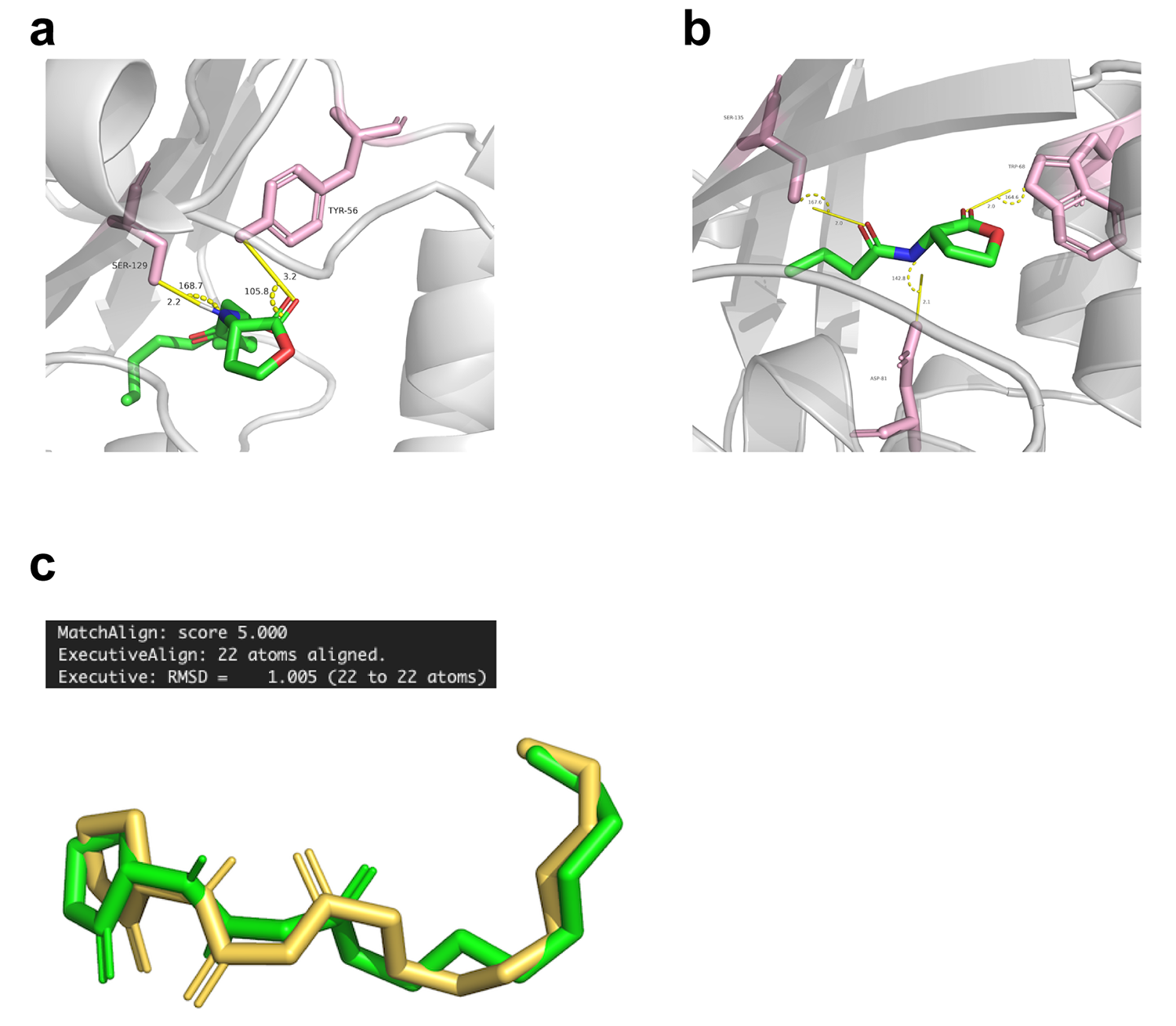
**

**Figure S1. a** and **b** The docked complex of QS receptors LasR and RhlR with its natural signal molecules and the visualizations of interactions in binding sites. **c** Docking protocol validation, C12-HSL redocked with LasR , and RMSD calculation (1.005 Å).

**
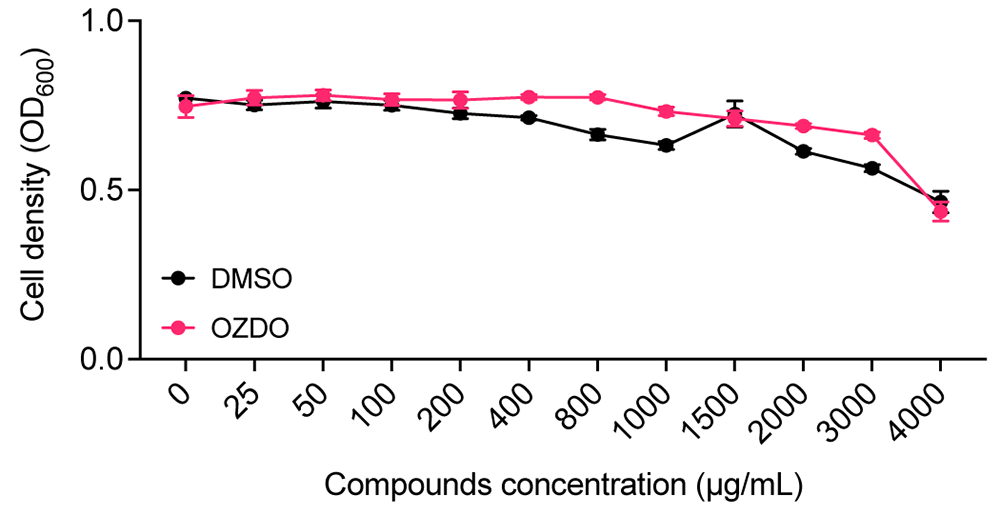
**

**Figure S2.** The minimal inhibitory concentration of OZDO test.


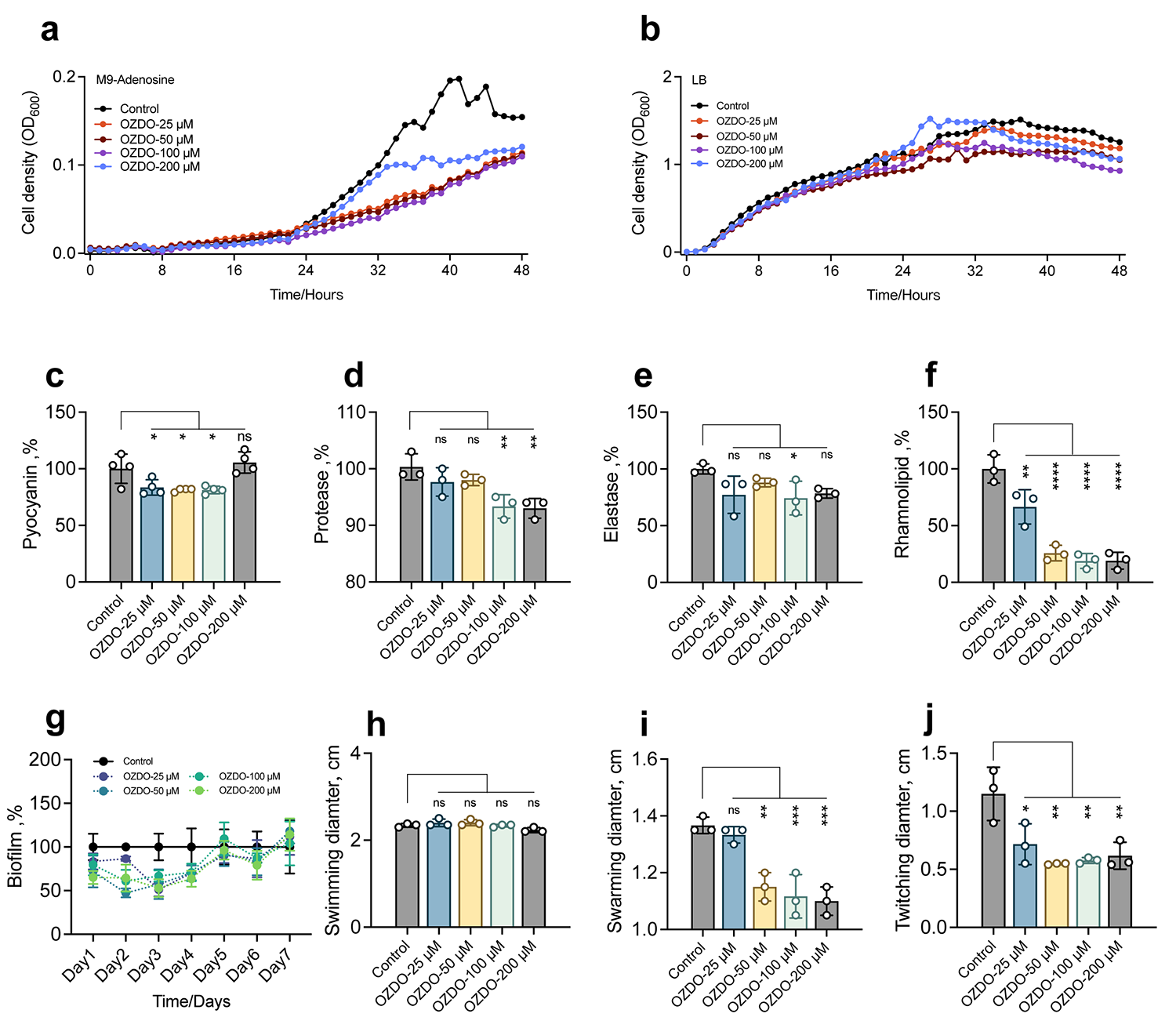


**Figure S3.** Phenotypic assay of OZDO (25 µM, 50 µM, 100 µM and 200 µM) on PA14. **a** and **b** Growth curve of PA14 with OZDO in M9-adenosine and LB. **c** Pyocyanin assay. **d** Protease assay **e** Elastase assay. **f** Rhamnolipid assay. **g** 7-day biofilm formation assay. **h** Swimming motility assay of. **i** Swarming motility assay. **j** Twitching motility assay Data shown were means ± SD of three independent replicates. One way ANOVA test compared to the control group. Statistical significance determined *means *P* < 0.05, **means *P* < 0.01, ***means *P* < 0.001, ****means *P* < 0.0001 and ns means not significant.

**Figure S4.** The protocol for synthesizing the compound OZDO. The synthetic method was referred to the previous description. 3-(benzo[d][1,3]dioxol-4-yl)oxazolidin-2-one: A suspension of oxazolidin-2-one (435.4 mg, 5.0 mmol), 4-bromobenzo[d][1,3]dioxole (1005.1 mg, 5.0 mmol), CuI (95.2 mg, 10 mol %), (S,S)-1,2-diaminocyclohexane (114.1 mg, 20 mol %), K_2_CO_3_ (1382.1 mg, 10 mmol) in anhydrous 1,4-dioxane (2.5 mL) was stirred in Schlenk tube under nitrogen at 110 °C for 16 h. At ambient temperature, the reaction mixture was quenched with NH_4_Cl (aq) (10 mL) and extracted with EtOAc (3 × 25 mL). The combined organic layers were dried over anhydrous Na_2_SO_4_. After filtration and evaporation of the solvents in vacuo, the crude product was purified by column chromatography on silica gel (Petroleum ether/EtOAc:) to yield the product (725 mg, 70 %) as a white solid. The purity was 97.50% (HPLC analysis, detection wavelength of 269 nm).

**Supplementary Tables**

**Table S1**. Primers used in this study

| Gene | Sequence (5’-3’) |
| --- | --- |
| *lasB*rtF | ATCGGCTACGACATCAAGAAGG |
| *lasB*rtR | CCGCTGTTGTAGTTGCTGGTG |
| *lasR*rtF | CTTCATCGTCGGCAACTAC |
| *lasR*rtR | GTCTGGTAGATGGACGGTTC |
| *rhlA*rtF | ACTGAACCAGGCGATGCTC |
| *rhlA*rtR | GCTCCAGGCAAGCCAAGTA |
| *rhlR*rtF | GCTCCTCGGAAATGGTGGT |
| *rhlR*rtR | GGAAAGCACGCTGAGCAAAT |
| *pqsArtF* | GCTGAGCGGTCCTTTGGC |
| *pqsArtR* | TGGAACCCGAGGTGTATTGC |
| *pqsR*rtF | CACTGGTTGAAGCGGGAGA |
| *pqsR*rtR | TCGTTCTGCGATACGGTGAG |
| *phzArt*F | GCAACTGGACCACGGAAAG |
| *phzArt*R | GCACGCAGTTTCTGTATCGG |
| *hcnArt*F | GCAGACATGACCATCCACCTC |
| *hcnArt*R | CGGTTGCTTTCGGTTTCCA |
| *16Srt*F | TCGCATCCTGTTGTCCTCCA |
| *16Srt*R | TTAGCCAGGGTCAGCGTCA |

**Table S2**. Susceptibility of *P. aeruginosa* PAO1 and clinical isolates to commonly used antibiotics (minimal inhibitory concentration, μg/mL).

| Strain | PAO1 | 7-61-28 | 7-R4-24 | 3-100-1 |
| --- | --- | --- | --- | --- |
| Aztreonam | 4 | 8 | 16 | 1 |
| Polymyxin B | 2 | 4 | 2 | 2 |
| Levofloxacin | 0.25 | 0.25 | 1 | 2 |
| Amikacin | 0.5 | 0.5 | 1 | 0.25 |
| Ciprofloxacin | 0.125 | 0.25 | 0.25 | 1 |
| Tobramycin | 0.25 | 0.5 | 0.25 | 0.25 |
| Gentamicin | 0.5 | 0.5 | 0.5 | 0.25 |
| Piperacillin | 4 | 4 | 16 | 4 |
| Cefepime | 2 | 4 | 2 | 2 |
| Cefotaxime | 16 | 8 | 8 | 2 |
