# Supplementary figures and images for: Multifaceted quorum-sensing inhibiting activity of 3-(Benzo[d][1,3]dioxol-4-yl)oxazolidin-2-one mitigates Pseudomonas aeruginosa virulence
Source: Virulence. 2025 Mar 19;16(1):2479103. doi: 10.1080/21505594.2025.2479103 (PMC12915424; doi:10.1080/21505594.2025.2479103)

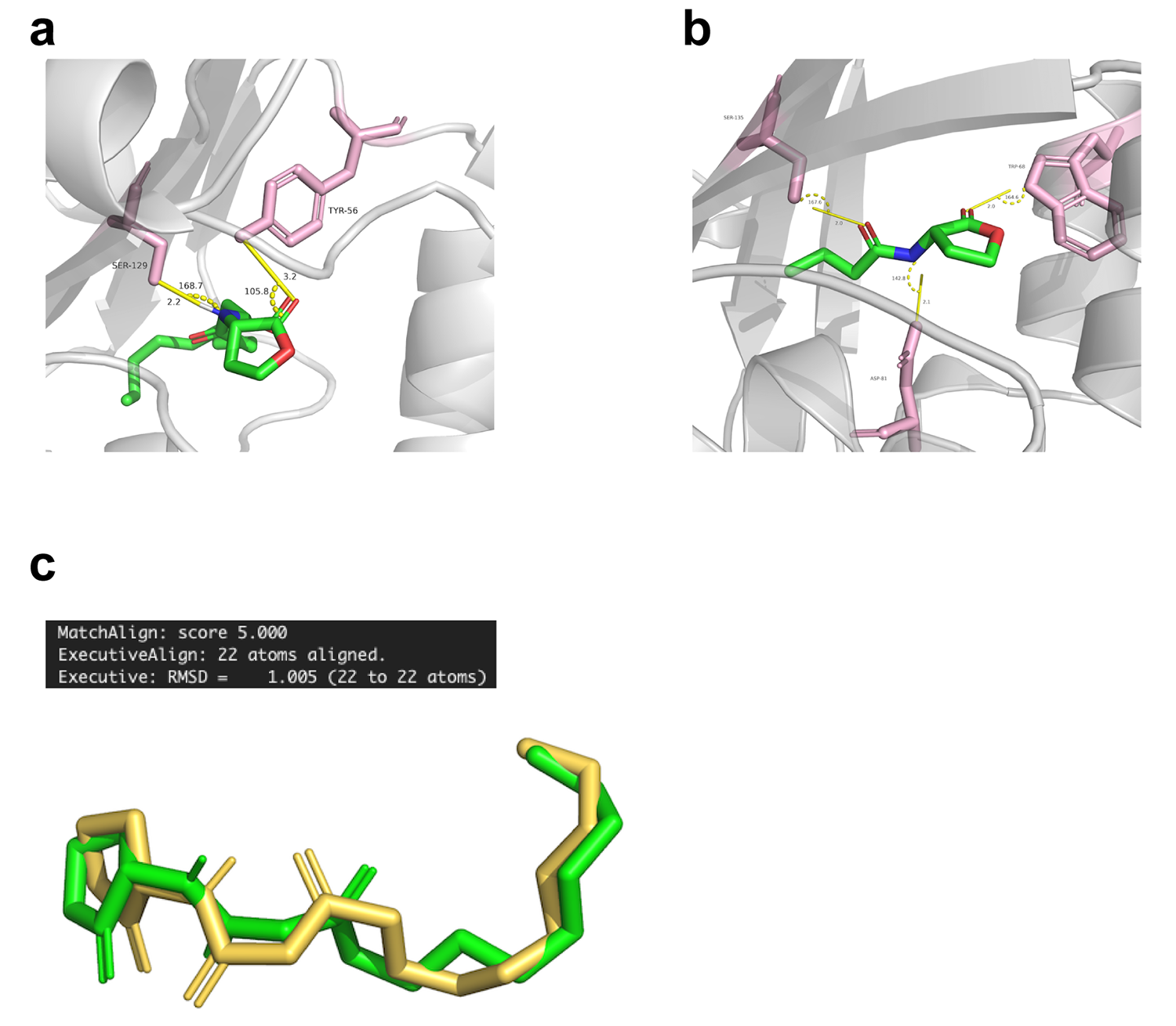

Supplement: Figure S1.tif [file KVIR_A_2479103_SM7654.tif]

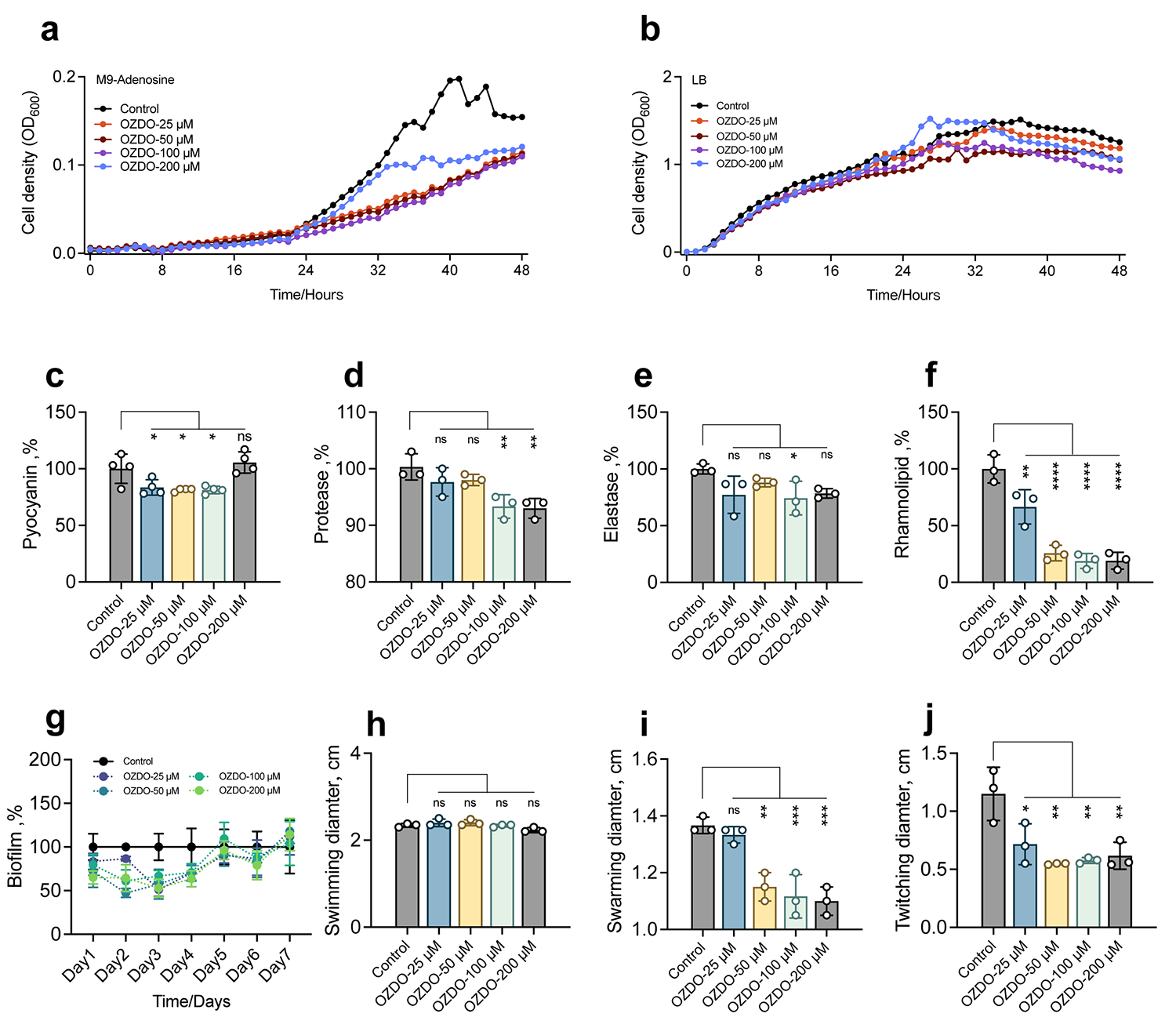

Supplement: Figure S3.tif [file KVIR_A_2479103_SM7653.tif]

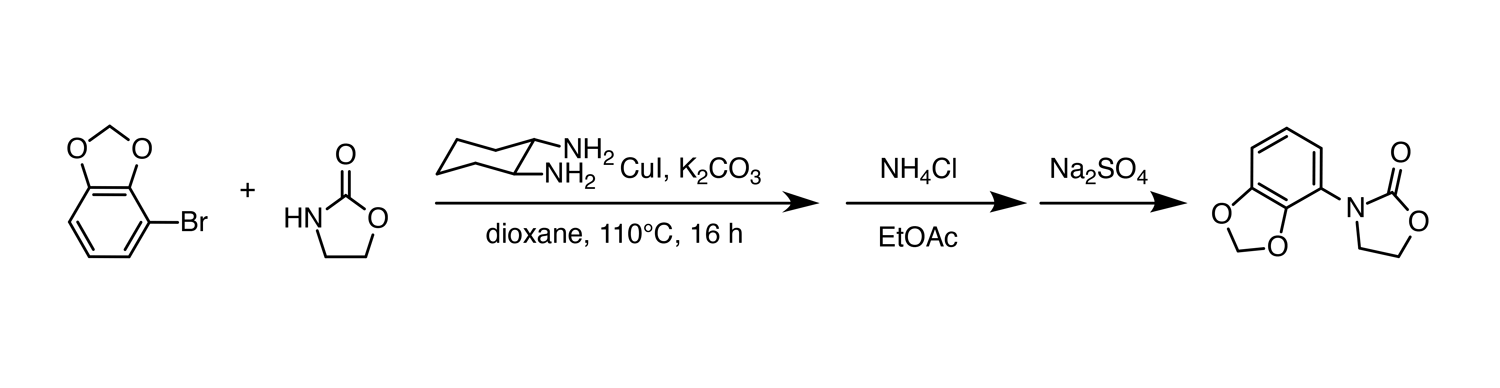

Supplement: Figure S4.tif [file KVIR_A_2479103_SM7652.tif]

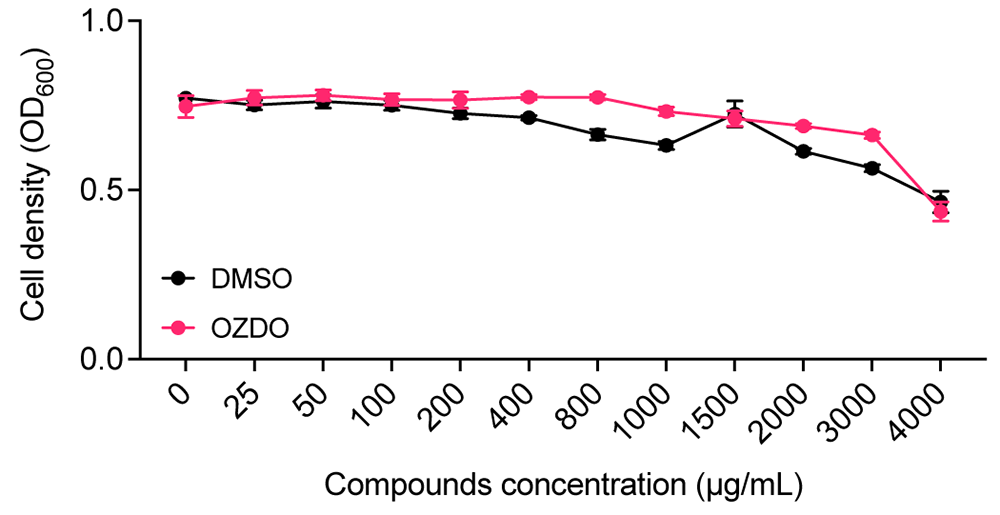

Supplement: Figure S2.tif [file KVIR_A_2479103_SM7650.tif]
