# Supplementary material for: Multifaceted quorum-sensing inhibiting activity of 3-(Benzo[d][1,3]dioxol-4-yl)oxazolidin-2-one mitigates Pseudomonas aeruginosa virulence
Source: Virulence. 2025 Mar 19;16(1):2479103. doi: 10.1080/21505594.2025.2479103 (PMC12915424; doi:10.1080/21505594.2025.2479103)
Supplement: Table S2.docx [file KVIR_A_2479103_SM7651.docx]

**Multifaceted Quorum-sensing Inhibiting Activity of 3-(Benzo[d][1,3]dioxol-4-yl)oxazolidin-2-one Mitigates** ***Pseudomonas aeruginosa* Virulence**

Yi Wu, Fulong Wen, Shiyi Gou, Qiman Ran, Yiwen Chu, Wenbo Ma*, Kelei Zhao*

Antibiotics Research and Re-evaluation Key Laboratory of Sichuan Province, School of Pharmacy, Chengdu University, Chengdu 610106, Sichuan, China

* Correspondence:

Kelei Zhao, Email: zhaokelei@cdu.edu.cn

Wenbo Ma, Email: mawenbo@cdu.edu.cn

**Supplementary Tables**

**Table S2**. Susceptibility of *P. aeruginosa* PAO1 and clinical isolates to commonly used antibiotics (minimal inhibitory concentration, μg/mL).

| Strain | PAO1 | 7-61-28 | 7-R4-24 | 3-100-1 |
| --- | --- | --- | --- | --- |
| Aztreonam | 4 | 8 | 16 | 1 |
| Polymyxin B | 2 | 4 | 2 | 2 |
| Levofloxacin | 0.25 | 0.25 | 1 | 2 |
| Amikacin | 0.5 | 0.5 | 1 | 0.25 |
| Ciprofloxacin | 0.125 | 0.25 | 0.25 | 1 |
| Tobramycin | 0.25 | 0.5 | 0.25 | 0.25 |
| Gentamicin | 0.5 | 0.5 | 0.5 | 0.25 |
| Piperacillin | 4 | 4 | 16 | 4 |
| Cefepime | 2 | 4 | 2 | 2 |
| Cefotaxime | 16 | 8 | 8 | 2 |
